# The limits of normal approximation for adult height

**DOI:** 10.1038/s41431-021-00836-7

**Published:** 2021-03-04

**Authors:** Sergei A. Slavskii, Ivan A. Kuznetsov, Tatiana I. Shashkova, Georgii A. Bazykin, Tatiana I. Axenovich, Fyodor A. Kondrashov, Yurii S. Aulchenko

**Affiliations:** 1 Skolkovo Institute of Science and Technology, Moscow, Russia; 2 Novosibirsk State University, Novosibirsk, Russia; 3 Moscow Institute of Physics and Technology, Moscow, Russia; 4 Institute for Information Transmission Problems (Kharkevich Institute), Moscow, Russia; 5 Institute of Cytology and Genetics SB RAS, Novosibirsk, Russia; 6 Institute of Science and Technology, Vienna, Austria; 7 Kurchatov Genomics Center, Institute of Cytology and Genetics SB RAS, Novosibirsk, Russia; 8 PolyOmica, ‘s-Hertogenbosch, PA The Netherlands

**Keywords:** Quantitative trait, Heritable quantitative trait

## Abstract

Adult height inspired the first biometrical and quantitative genetic studies and is a test-case trait for understanding heritability. The studies of height led to formulation of the classical polygenic model, that has a profound influence on the way we view and analyse complex traits. An essential part of the classical model is an assumption of additivity of effects and normality of the distribution of the residuals. However, it may be expected that the normal approximation will become insufficient in bigger studies. Here, we demonstrate that when the height of hundreds of thousands of individuals is analysed, the model complexity needs to be increased to include non-additive interactions between sex, environment and genes. Alternatively, the use of log-normal approximation allowed us to still use the additive effects model. These findings are important for future genetic and methodologic studies that make use of adult height as an exemplar trait.

## Introduction

Since the dawn of biometry and genetics, human height has served as a model, exemplar quantitative trait. Studies of adult height have shaped a classical biometric and quantitative genetic approach to inheritance [1–3]. Nowadays, new quantitative genetic methods are often first applied to adult height, and the findings for height have profound effects on the ways we look at and study other quantitative and complex traits. Specifically, the studies of height have triggered the formulation of the problem of “missing heritability” [4–6] as well as have fuelled progress in understanding its sources [7, 8].

An important part of our view of height is an assumption of additivity of effects that is explicitly made in early works and is justified by a “physiological” argument: “stature is not a simple element, but a sum of the accumulated lengths or thicknesses of more than a hundred bodily parts” [1]. One should also remember that a large part of the appeal of the use of additive models to describe inheritance of quantitative traits lies in the fact that they have been demonstrated both theoretically and empirically to be parsimonious, that is, to have great explanatory power while being very simple [9–12].

Additivity implies normality, and the adult height serves as an empirical example of a normally distributed biological trait in textbooks on statistics [13–15]. Furthermore, an inheritance of human height is described by a model in which many genetic effects add up (additive polygenic model) [3, 5]. The dominant practice in human height genetics is to treat all effects as additive, and to analyse untransformed or linearly transformed height (see Supplementary Note 1), which implies an assumption of normality of the distribution of residuals.

Thus, from studies of adult height, we know that an “ideal” quantitative trait results from a sum of the individual contribution of different influences, and it has a normal distribution of residuals (perhaps, after some transformation) [16, 17]; adult height being the prime and convincing example of such trait.

At the same time, it is well-understood that additivity of effects and normal distribution of residuals is only an approximation to the distribution of height in human populations. Practically, we notice several peculiarities in the distribution of adult height (see Supplementary Note 2). One of them is apparent growth of standard deviation of height with the mean height across populations; another is possibly multiplicativity of the effects of sex. These observations may suggest the presence of non-additive interactions for human height. However, up until now, no evidence for non-additive genetic interactions was found for height [6, 18]. Moreover, if non-additive effects were to exist for polygenic traits, very large sample sizes would be required to detect them [9].

Here, we asked the question if we have now reached the stage where non-additive effects can be detected for the adult height. The assumption of additivity is intimately linked to the assumption of normality; therefore, we also explored the limits of normal approximation for the distribution of the residuals of adult height in a large diverse population.

We collected literature data and analysed the distribution of mean height and its variance across a wide range of populations. We also analysed how the mean and variance of height depends on sex, genes, and sociodemographic factors in 369,153 white British participants of the UK Biobank study [19].

## Materials and methods

### Changes of SD and CV with the mean height and changes of relative and absolute height difference between sexes in world populations (Fig. 1)

The regression analysis has been conducted between SD or CV and mean height of females from 54 developing countries, as reported by Subramanian et al. [20]. We excluded from consideration countries for which sample size was less than 1000 (Comoros) and for which observations were deviating by >3 SD from the overall average (Congo Dem. Rep., Congo Rep., Guatemala). The retained 50 populations were taken for further analysis. To avoid domination of a few very large samples, we assumed equal weight for observations coming from different populations.

**Fig. 1:**
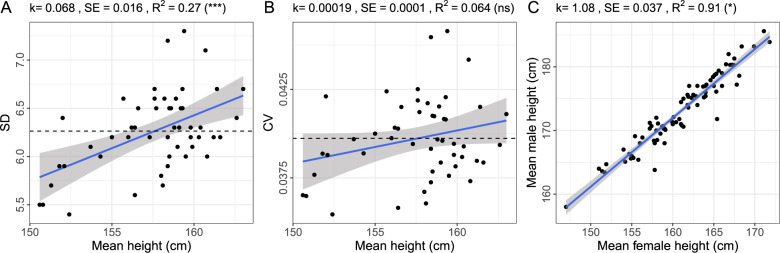
Relation between parameters of the distribution of adult human height across populations. Linear regression of standard deviation (**A**) and CV (**B**) of height on mean height of women from ref. [20]. The dashed line shows the overall mean. (**C**) Linear regression of mean male height on mean female height in populations from ref. [21]. Unweighted linear regression was used to estimate the trend (k), its standard error (SE), the adjusted R^2^ and, in brackets, the significance of deviation of the regression coefficient from zero for **A**, **B** and from one for **C** (*p* < 0.001–***; *p* < 0.01–*; *p* > 0.05—ns) (shown at the top of each panel).

We analyzed dependence between male and female average height in worldwide human populations using data from the internet resource [21]. The first stage of filtration included removing rows with missing data for male or female and then removing rows with values deviating from average value more than by 3 SD for corresponding sex. In the second stage of filtration, we excluded repeating data for the same countries and retained one survey result for each country and/or national group. The criteria of filtering were the following: if urban/rural and general population data were available, the general one was retained; if different age intervals were available, the wider one was retained; if data for several ages were available, the one closer to 21 was retained. Ethnic groups in one country were considered as separate populations. Eventually, 80 populations passed all the filters. The data used are presented in Supplementary Table 1; the data points passing our quality control are indicated.

### Analysis of UK biobank data

We analysed 369,153 white UK Biobank participants belonging to six groups defined by ethnic background and place of birth (Supplementary Table 2). We considered effects of sex, genotype, and residual effects. The genotype was included in analysis in the form of polygenic height score (PGHS), defined as the weighted prevalence of height-increasing alleles in the genotype. Factors related to socioeconomic status and study covariates were used to construct a single linear predictor, hereafter called the “residual predictor”. All three predictors were strongly associated with adult height. In the main text, we only reported *p* values for joint analysis of all data, with per-group results reported in Supplementary Tables.

#### Definition of studied groups and phenotypic quality control

We have restricted the analysis of UK Biobank (UKB) data [22] to individuals of European (white) descent whose samples were used to compute the genetic principal components (PCA cohort, UKB field 22020), thus excluding close (degree ≤3) relatives from our analysis. Within PCA cohort, we have defined groups based on self-reported ethnic background (field 21000), genetic ethnic grouping (“genetically Caucasian” field 22006; available only for self-reported ethnicity “white British”), and place of birth (field 1647—country of birth inside UK, and 20115—outside UK). In total, we ended up with six analysis groups (English, Scottish, Welsh, Other British, Irish, Other White), defined by place of birth and ethnic background (see Supplementary Table 2 for details). In the final dataset we only considered individuals with complete information on all of the following phenotypes and covariates: height, sex, income, year of birth, age at recruitment, assessment centre, genotype, genetic principal components, genotyping batch.

Each of the six analysis groups was stratified by sex; within these 12 sub-groups we have excluded individuals who deviated from the mean by 4.75 standard deviations or more (in a study of the size of UKB, under the null hypothesis this cut-off translates into expectation of one outlier). In total, 32 people were excluded according to this criterion. Supplementary Table 2 shows the final number of individuals in each analysis group.

Throughout this manuscript, we use “height” for height in centimeters, while “log-height” is used for the base-10 logarithm of height.

#### Polygenic height score (PGHS) computations

We considered 697 SNPs associated with height, reported in Wood et al. The SNPs were clustered in 423 loci, with a locus defined as one or multiple jointly associated SNPs located within a 1-Mb window [23]. From each locus, we have selected one SNP that demonstrated the lowest *p* value in the GIANT univariate analysis. We observed that rs9404952:G > A (NC_000006.11:g.29804165 G > A) was neither genotyped nor imputed in the UKB. Three SNPs—rs1420023:C > A,G,T (NC_000012.11:g.12876111 C > D), rs1659127:G > A,C,T (NC_000016.9:g.14388305 G > H), rs7567851:G > A,C (NC_000002.11:g.178684720 G > M)—were genotyped and imputed in UKB with alleles different between direct genotyping and imputations. We therefore excluded these three SNPs from analysis because of ambiguity. From the residual 419 SNPs we have selected 305 that satisfied all of the following criteria: had imputation quality in UKB greater than 0.9; had univariate *p* value less than 5 × 10^−10^ in GIANT analysis of Wood et al.; had EAF difference less than 5% between six above defined UKB groups and “250 K GWAS Meta-analysis” of Wood et al. [23]. To load BGEN-encoded genotype data into the R environment ‘rbgen’ package [24] was utilised. We used univariate estimates of effect sizes reported by Wood et al. to calculate weighted PGHS. PGHS was then centred and scaled (so that mean is zero and variance is unity) in each of the six analysis groups defined above.

For the purposes of analysing epistasis we also constructed an “updated” PGHS based on 712 loci (3290 SNPs) reported in Yengo et al. [25]. That study of Yengo et al. was conducted with UKB data; all SNPs were present in our data, had imputation quality >0.3 and MAF > 0.0001 in the UKB, and univariate association *p* < 10^−8^ in the Yengo analysis. We used the same procedure, as described above, to load data and define a polygenic score.

Although GWAS conducted by Yengo et al. [25] is more powerful than that reported by Wood et al. [23], the former includes UK Biobank participants. This may lead to upward bias in the proportion of variance explained by the PGHS. In principle, our conclusions do not directly depend on an absolute value of the proportion of variance explained. Still, we decided to use PGHS constructed from the GWAS results of Wood et al. [23] for most analyses, to avoid potential bias. We used PGHS constructed from the GWAS results of Yengo et al. [25] in our supportive analyses to demonstrate that with increased score power the epistatic effects become more pronounced, as predicted by our main analysis.

#### Computation of residual predictor of height

Using data on 370 thousand white British individuals we estimated a linear mixed model of height that included sex, PGHS, year of birth, year of visit to the assessment centre (five levels), household income, analysis group (six levels) and ten first principal components of genetic variation as fixed effects and assessment centre code and genotyping batch as random effects. From the predicted height we then have subtracted estimated effects of sex and PGHS, after which the resulting predictor was centred and scaled to have mean zero and variance of one. The linear mixed model was estimated using the R package “lme4” [26].

#### Analysis of scaling of effect sizes and standard deviation (Figs. 2 and 3)

**Fig. 2:**
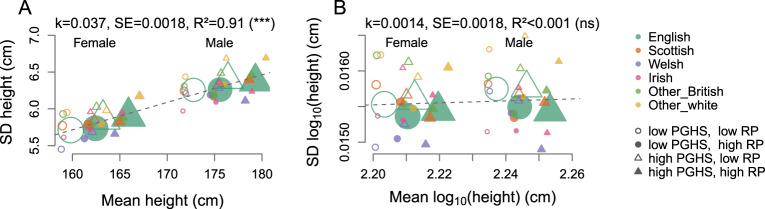
Changes of SD with the mean height and log-height in UK Biobank. Relation of standard deviation to mean of height (**A**) and log-height (**B**) for six groups of British individuals of white descent from UK Biobank, defined based on place of birth and split by sex, median polygenic score, and median residual predictor (48 groups in total). The size of a symbol is proportional to the regression weight, defined as twice the group size. Weighted linear regression was used to estimate the trend (k), its standard error (SE), the adjusted R^2^ and, in brackets, the significance of deviation of the regression coefficient from zero (*p* < 0.001–***; *p* > 0.05—ns) (shown at the top of each panel).

To generate Fig. 2, we divided each of the six analysis groups into eight sub-groups defined by sex, high/low polygenic score, and high/low residual predictor. In total, we obtained 48 (6 × 2 × 2 × 2) groups of individuals. In each group, we estimated the effect of mean height (mean log-height) on corresponding standard deviation by linear regression model with weights, defined as the group size.

**Fig. 3:**
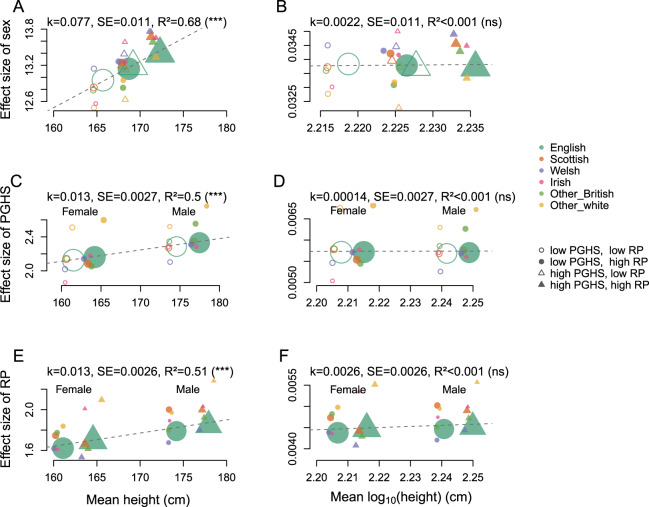
Changes of the effects of different factors with the mean height and log-height in UK Biobank. Relation between the estimate of the effect size of sex (**A**, **B**), genotype (**C**, **D**; genotype was defined as a polygenic height score, PGHS), other factors (**E**, **F**; a linear residual predictor, RP, combining sociodemographic and study covariates) and mean height (**A**, **C**, **E**) and log-height (**B**, **D**, **F**) for six groups of British individuals of white descent from UK Biobank, defined based on place of birth. The six groups are additionally split by sex (**C**–**F**), median polygenic height score (**A**, **B**, **E**, **F**), and median residual predictor (**A**–**D**). The size of a symbol is proportional to the group size (used as the regression weight). Weighted linear regression was used to estimate the trend (k), its standard error (SE), the adjusted R^2^ and, in brackets, the significance of deviation of the regression coefficient from zero (*p* < 0.001–***; *p* > 0.05—ns) (shown at the top of each panel).

Within each of the six analysis groups we computed median polygenic score and median residual predictor. To generate Fig. 3, we estimated effect of a specific factor (sex, polygenic score, and the residual predictor) on height and log-height within six ethnic groups, additionally sub-divided by the two other factors. For example, we calculated the effect of sex on height (log-height) in six analysis groups additionally sub-divided by median polygenic score and the median residual predictor. In total, we considered 24 sub-groups for each factor. Within each subgroup, an effect of a factor on height (log-height) was estimated using a univariate linear regression. We applied a weighted linear regression model to study the relation between mean height (mean log-height) and the effects of studied factors. Weights were defined as the group size.

#### Epistatic model

We formalise the additive models as

$$Height = \mu + \beta _1PGHS + \varepsilon = \mu + \beta _1(a_1g_1 + a_2g_2 + \ldots + a_ng_n) + \varepsilon$$

where *PGHS* is the vector of PGHS values, *g*_1_,…,*g*_*n*_ are the vectors of genotypes of loci included in *PGHS*, *μ* is an intercept and *β*_1_ is a scaling factor (both estimated from the data) and *a*_1_, …, *a*_*n*_ are the weights fixed at the values corresponding to the estimates of additive effects obtained by previously published studies.

The epistatic model is formalised as

$$Height =	\; \mu + \beta _1PGHS + \beta _2PGHS^2 + \varepsilon \\ =	\; \mu + \beta _1\left( {a_1g_1 + a_2g_2 + \cdots + a_ng_n} \right)\\ 	+ \beta _2\left( {a_1g_1 + a_2g_2 + \cdots + a_ng_n} \right)^2 + \varepsilon ,$$

Thus our epistatic model only considers the dominance and pairwise epistatic interactions and estimates these effects through a single parameter, *β*_2_. This is made possible by assuming that all pairwise interaction effects are proportional to the products of the published additive genetic effects from the linear model.

#### Comparison of the variance explained by additive and multiplicative models

To compare the proportion of variance explained by the additive and multiplicative models we fitted a linear model for height and log-height on each analysis group. The model included PGHS, sex, and the variables that were used to define RP. Briefly, two linear regression models were fitted:

$$Height =	\; \mu + \beta _1Sex + \beta _2PGHS + \beta _3RP_1 + \beta _4RP_2 \\ 	+ \ldots + \beta _{18}RP_{16} + \varepsilon$$

$$\log _{10}\left( {Height} \right) =	\; \mu + \beta _1Sex + \beta _2PGHS + \beta _3RP_1 + \beta _4RP_2 \\ 	+ \ldots + \beta _{18}RP_{16} + \varepsilon$$

where *Height*, *Sex*, *PGHS* are vectors of height, sex and PGHS values correspondingly, *RP*_1_ to *RP*_16_ are the vectors of the residual predictor components as described above, *μ* and *β*_1_ to *β*_18_ are the corresponding regression coefficients, and ɛ is the vector of residuals. The proportion of variance of height explained by the additive model was estimated as the relative decrease in the variance of height after subtracting observed from the values predicted by the additive model. The proportion of variance of height explained by the multiplicative model was estimated as the relative decrease in the variance of height after subtracting observed from the exponentiated value of the predicted log-height (see Supplementary Table 3).

#### Testing type 1 error in potential GWAS

For minor allele frequencies 0.01, 0.05, 0.1, 0.5 we generated random vectors of genotypes (0, 1 or 2) distributed according to Hardy–Weinberg equilibrium. We then tested the association of these random genotypes with the residuals of height and log-height, obtained for 369,153 individuals of European (white) descent. The residuals were obtained from a regression model including sex, PGHS, and RP as independent variables and the statistical strength of association was characterised by the *Z*-test (the ration between estimated regression coefficient and its standard error). For each allele frequency 1 million independent simulations were performed. The equality of distributions of *Z*-test statistics obtained for height and log-height was tested with Kolmogorov–Smirnov test.

#### Transferability of prediction between groups of different origin

To analyse the transferability of height prediction models between groups of different origin we generated linear height and log-height prediction models using British people of white descent born in England as a training set. The models included sex, PGHS, income (“Average total household income before tax”, UKB field 738) and age (“Age when attended assessment centre”, UKB field 21003) as independent variables. Groups where height and log-height were predicted were defined either as described above (Scottish, Welsh, Other British, Irish, Other white) or defined by Ethnic background (UKB field 21000). Supplementary Table 4 provides details of these groups.

#### Other statistical analyses

We used the *lm* function from R package ‘stats’ [27] to fit linear models with height or logarithm of height as the response variable. To compare variance between two groups we used Levene’s test [28] as implemented in R package ‘car’ v.2.1–6 [29].

For ratios, the confidence interval (CI) was computed using Fieller’s theorem [30]. The test of significance between two ratios was performed using the mean difference test, with standard deviations of the estimate of a ratio computed using delta-method [17].

## Results

First, we reviewed anthropological literature concerned with the study of the distribution of height in different populations and collected the data on the mean height, its standard deviation, and sample size where it was possible. Across different populations, we studied how the mean height relates to the SD and CV and analysed effects of sex.

In the analysis of 50 populations [20] we observed a scaling of standard deviation (SD) with the mean female height—that is, the variation of height becomes larger as the average height of a population increases (Fig. 1A). At the same time, the CV does not display this behaviour to a significant extent, being low and rather stable across different populations (see Fig. 1B). The mean height explained 27% of the variance of the SD (*p* < 0.001), while only 6% of the variance of the CV (*p* = 0.08).

To determine whether male and female mean height are related multiplicatively or additively, we regressed the mean male height on the mean female height (Fig. 1C). We used the data on the mean male and female height coming from a large number of populations from the literature [21]. The intercept from the linear regression model was −0.77 and did not significantly differ from zero (*p* = 0.9). The slope in this model was 1.080 that differs significantly from 1 (*p* = 0.003). This observation supports multiplicative contribution of sex to the height variance.

Thus, when we study a range of diverse human populations, both SD and effects of sex scale up with increased mean—a behaviour, not expected from a normal, but common for a log-normal distribution [31].

Next, we used data from the UK Biobank [22] to confirm and extend the above results. We considered three strong predictors of height, namely, sex, genotype (PGHS summarising effects of 305 SNPs from ref. [23]), and residual effects (a linear residual predictor, combining socioeconomic and study covariates). In a variety of groups of British individuals of white descent, defined by place of birth, sex, genotype and the value of residual predictor, we confirm that the SD increases with the group mean (Fig. 2A). The variance of height was significantly higher in men (*p* < 10^−100^ for English, *p* < 10^−50^ for all-but-English), in people with higher PGHS (*p* = 10^−16^ for English, and *p* = 7 × 10^−8^ for all-but-English) and those with higher value of the residual predictor (*p* = 2 × 10^−9^ for English, and *p* = 10^−3^ for all-but-English) (Supplementary Table 5A).

We also confirm previous observation that the effects of sex increase with increased group’s mean (Fig. 3A). Moreover, we see similar increase for the effects of the polygenic score (Fig. 3B) and for the residual predictor (Fig. 3C).

Above big data observations contradict the traditional practice of modelling the height with the mean-model. In this model, effects sum up and the residuals are distributed normally with a fixed standard error. The changes in SD and effect sizes, while being small (we see an increase of a few millimetres per one decimetre change in the mean), are clear and significant.

We next have used individual-level UK Biobank data to explore all pairwise interactions between sex, polygenic score, and the residual predictor. The interactions were introduced into the model as a product between corresponding variables, for example, the interaction between sex (coded as 0 for female and 1 for male) and PGHS was defined as sex*PGHS. We observed significant and replicable interactions between all three factors (see Supplementary Table 6A). In the model including all three interactions, we established the significance of each interaction effect by testing its deviations from zero (Wald test). The interaction between sex and polygenic score was the strongest (*p* = 10^−12^ for English, and *p* = 4 × 10^−6^ for all-but-English), the sex by residual predictor interaction was next significant (*p* = 7 × 10^−12^ for English, and *p* = 10^−5^ for all-but-English), and interaction between polygenic score and the residual predictor was weaker although still significant (*p* = 0.01 for English, and *p* = 0.001 for all-but-English). We have also tested for the effects of epistasis. In quantitative genetics, the term “epistasis” implies any deviation from additivity (on the appropriate scale) of genetic effects of different loci [16]. PGHS corresponds to the sum of additive genetic effects. Although epistatic interaction may be presented by a function of any kind, here we examine the deviation from purely additive genetic effects expressed through squared PGHS. The pairwise interactions between alleles are described with a single parameter under an assumption that the coefficients of pairwise interactions are proportional to the product of additive effects. This epistatic model is described in more detail in the Materials and methods section. In a model also including sex, residual predictor, and the main effects of polygenic predictor, the effect of squared polygenic score was significant in English but could not be replicated (*p* = 1.32 × 10^−2^ for all, *p* = 2.02 × 10^−2^ for English, and *p* = 0.346 for all-but-English). We speculated that our polygenic score is probably too weak to detect epistasis, and updated it to include 3290 SNPs from 712 loci [25]. Indeed, the epistatic effects of the updated polygenic score were stronger and were replicated (*p* = 4 × 10^−7^ for all, *p* = 2 × 10^−4^ for English, and *p* = 2 × 10^−4^ for all-but-English).

The observations of increased effects of various predictors and of the SD with the mean, the effects of interactions, and variance heterogeneity we observed for adult height lead us to try an alternative, log-normal, approximation (see Supplementary Note 3 for details, and see Supplementary Note 4 for discussion of other non-linear transformations). The logarithm of a log-normally distributed character is distributed normally, and the effects that multiply on the original scale, add up on logarithmic. Indeed, for the log-height we observe that the effects added up, and we observed an absence of scaling of the SD (Fig. 2B) and of the effects (Fig. 3B, D, E) with the mean, absence of significant effects of interactions (Supplementary Table 6B), and absence of detectable variance heterogeneity (Supplementary Table 5B). Also the epistatic effects, defined as the effects of the squared polygenic score, were not significant for the log-height (*p* = 0.76 for all, *p* = 0.67 for English, and *p* = 0.91 for all-but-English; for the updated polygenic score, *p* = 0.48 for all, *p* = 0.89 for English, and *p* = 0.09 for all-but-English). Thus, logarithmic transformation both stabilises the variance and allows us to use a simpler model for the mean.

Finally, we addressed the question of how well the normal and log-normal distributions approximate population distribution of height. Overall, the multiplicative model describes the data only slightly better, and the gain in the proportion of variance explained is minimal (less than 0.1%, see Supplementary Table 3). The study of quantile-quantile plots (Supplementary Fig. 1) shows that the normal distribution does not approximate the tails of height distribution well. The log-normal distribution does better for the right tail, although it cannot accommodate the left tail, where, compared to the expectation, we observe too many short people.

Furthermore, we explored how well height predictive models trained in people born in England translate to people born elsewhere (Supplementary Table 4). Overall, there was a tendency for the multiplicative model to transfer better than the additive model: for 9 out of 13 groups studied, the proportion of variance explained by the log-height model was higher, as well as the median bias was smaller. However, this frequency was not significantly deviating from the null 50:50 expectation (*p* > 0.05).

The multiplicative model of height predicts that at the extremes, people will be taller than predicted by an additive model, while in the centre, the additive model will overestimate the height. When we compare the observed distribution of height with the normal and log-normal, we observe that with a single exception both approximations provide an excellent fit to the data (Supplementary Fig. 2, Supplementary Table 7). The notable exception is the prediction of male height in the middle of the distribution; in accordance with the expectation from the multiplicative model, the normal approximation overestimates the height (by almost 1 mm, nominal *p* < 0.001, Bonferroni-corrected *p* = 0.03).

## Discussion

Here, we analysed adult height using summary-level data from a variety of world populations and big individual-level UK Biobank data. We demonstrated that when the height of hundreds of thousands of individuals is analysed, the model complexity needs to be increased to include non-additive interactions between sex, environment and genes. Alternatively, the use of log-normal approximation allowed us to still use the additive effects model. Thus, our analysis suggests that the log-normal approximation may be a useful alternative to the normal approximation in analysis of big (hundreds of thousands of individuals) height data, analysis of heterogeneous populations, and analysis of the extremes of height. These findings are important for future genetic and methodologic studies that make use of adult height as an exemplar trait.

Several independent lines of evidence support our conclusion about the presence of multiplicative effects and potential role of log-normal approximation for height. For example, the fact that variation of height is different between human populations, while the coefficient of variation is rather stable, has been documented in socioeconomic and anthropological literature [32, 33], leading some social scientists and economists to postulate a log-normal distribution of height [32] between populations. From the biological perspective, at least for species other than humans, the growth of organisms is believed to be a multiplicative process [34].

Our findings may be discussed in context of measurement theory [35]. This theory suggests that theoretical context determines the scale type of measurements and which transformations of those measurements can be made without compromising their meaningfulness. In the case of human height, as conventionally measured, theoretical context [34, 35] as well as results of this work may suggest a “ratio” scale (using terminology of [35]), under which a statement such as “on average, men are 8% taller than women” is meaningful.

As we detailed in the Introduction, prehaps, with the exception of sex, the multiplicative effects of other factors, such as genes and environment, on height were not appreciated before. We believe that this may be explained by the fact that the multiplicative effects are very small and their discovery asked for the sample sizes that were out of reach until very recent. In contrast to the traits that are well-known to be distributed log-normally (e.g. weight, body mass index, triglyceride level, and many others), the within-population coefficient of variation of adult height is small (CV of 3–5%, see, e.g. [36])—as soon as the large effect of sex is dealt with in an appropriate manner. When CV is small, and the effects in question are small, the additive and multiplicative models are almost indistinguishable, and normal and log-normal distributions both fit the data well [37]. This small deviation from additivity is hard to detect unless very large sample sizes are employed. Until recently, studies of individual-level height were restricted to studies of just a few thousands of individual measurements. Only availability of individual-level epidemiological and genetic data on hundreds of thousands of individuals in the UK Biobank made it possible for us to determine the multiplicative interactions between sex, genes, and the environment.

Although given the small CV the normal and log-normal approximations of height may be almost equivalent, still, some differences in statistical properties may be expected. Indeed, the use of log-normal approximation improves the model fit to the data, although it does that by very little (the difference in the proportion of variance explained is less than 0.1%). The predictions from the two models are also essentially the same (see Supplementary Table 8). The transferability of prediction from one population to another is somewhat better for the multiplicative model (Supplementary Table 4), but this finding is not significant. In terms of distribution of type 1 error under the null hypothesis, the uses of both normal and log-normal approximations are equivalent (Supplementary Table 9).

What may be expected in the future, when many millions of samples could be analysed? From our analysis it follows that while the use of log-normal approximation is expected to lead to better prediction, on population level the gain is very small. At the same time, deviation may be pronounced for individuals at the edges of distribution. We do not expect that the use of either normal or log-normal approximation will lead to large differences in type 1 or type 2 error. However, with progressively larger sample sizes the epistatic effects will become detectable for individual variants. At the moment, under normal approximation, we can already detect the gene by sex interaction for the lead SNP rs143384:G > A (*p* value for interaction is 0.037). Assuming that the distribution of height in mega-cohorts is better approximated by the log-normal rather than normal distribution, with even larger sample sizes, both Gene by Sex as well as Gene by Gene interactions will become significant for genetic variants with progressively smaller effects. Hence, if one were to keep the model simple, one would likely have to turn to log-normal approximation.

The question whether one should use normal or log-normal approximation to study human height depends much on the scientific question being asked. As we demonstrated, for prediction of means, as well as in the context of heritability estimation and GWAS we expect that differences are negligible. However, if one aims to study interactions and aims to predict variation of height, the difference between using normal and log-normal approximation will be substantial.

Thus, as far as we can see, our discovery mainly has a conceptual value and may guide better interpretation of the results of analysis of big data. As we demonstrate, a study of big height data under an additive model demonstrated variance heterogeneity and interactions. Variance heterogeneity across genotypes is often interpreted as evidence of possible interactions [25–27] or environmental sensitivity [28]; and each specific (e.g. gene by sex, gene by environment, gene by gene) interaction tend to be explained in terms of the underlying biology. In case of height, though, using log-normal instead of normal approximation allows using a simple parsimonious model that assumes that the effects add (on the log-scale).

On a side note, we would like to reflect on the fact that most anthropometric traits, such as height, weight, body mass index, are well approximated by a log-normal distribution with male having greater mean and hence greater variance. This may be one of the explanations of the “greater male variability” in physical characteristics, first noted by Darwin: “several studies had been conducted to demonstrate that variability was indeed more characteristic of males … The biological evidence overwhelmingly favoured males as the more variable sex … The cause of the greater general variability in the male sex, than in the female is unknown” [38].

To conclude, we analysed adult height using summary-level data from a variety of world populations and big individual-level UK Biobank data. Our analysis shows that when the height of hundreds of thousands of adults is analysed, the non-additive interactions can be detected. This leads to increased model complexity and a “breakdown” of the classical example of the classical quantitative genetics. This apparent breakdown could, however, be avoided, and the additivity assumption could be kept if the log-normal approximation is used. We speculate that future increase in the volumes of available data will eventually force us to further review the assumptions about distribution of adult height and the model of its control.

Our findings are important for the field of quantitative and complex trait genetics, because new methods are often tested using adult height, and the findings for height are often extrapolated and have profound effects on the ways we look at and study other traits.

## Supplementary information

Supplementary Material

Supplementary Tables

## Data Availability

This study makes use of genotype and phenotype data from the UK Biobank data under project # 41601, “Non-additive effects in control of complex human traits”. UKB data can be accessed upon request once a research project has been submitted and approved by the UKB committee. Results of model estimation and summary statistics are available as Supplementary Tables to this manuscript and also in Zenodo repository with the following 10.5281/zenodo.4115911.

## Code availability

The custom code used in the manuscript is available at GitHub (https://github.com/ivkuz/HumanHeight.git).
