# Supplementary material for: The limits of normal approximation for adult height
Source: Eur J Hum Genet. 2021 Mar 4;29(7):1082–91. doi: 10.1038/s41431-021-00836-7 (PMC8298501; doi:10.1038/s41431-021-00836-7)
Supplement: Supplementary file 1 — Supplementary Material [file 41431_2021_836_MOESM1_ESM.pdf]

Supplementary materials

to the manuscript "The limits of normal approximation for adult height"

by Sergei A. Slavskii[1,2,3]*, Ivan A. Kuznetsov[1]*, Tatiana I. Shashkova[2,3,4], Georgii A. Bazykin[1,4], Tatiana I. Axenovich[5], Fyodor A. Kondrashov[6], Yurii S. Aulchenko[2,3,5,7,8]**

[1] Skolkovo Institute of Science and Technology, Moscow, Russia

[2] Novosibirsk State University, Novosibirsk, Russia

[3] Moscow Institute of Physics and Technology, Moscow, Russia

[4] Institute for Information Transmission Problems (Kharkevich Institute), Moscow, Russia

[5] Institute of Cytology and Genetics SB RAS, Novosibirsk, Russia

[6] Institute of Science and Technology, Vienna, Austria

[7] Kurchatov Genomics Center, Institute of Cytology and Genetics SB RAS, Novosibirsk, Russia

[8] PolyOmica, 's-Hertogenbosch, the Netherlands

* These authors contributed equally to this work

** Correspondence: yurii@bionet.nsc.ru

Table of content

Supplementary materials **1**

Table of content 2

Supplementary notes 3

Supplementary Note 1. Historical and contemporary studies assume that the effects on adult height sum up. 3

Supplementary Note 2. Peculiarities in the current model of height. 5

Supplementary Note 3. Normal and log-normal approximations for height. 8

Supplementary Note 4. Distribution and variance-stabilising transformation of big height data. 12

Supplementary figures 15

Supplementary Figure 1. 15

Supplementary Figure 2. 16

References 17

Supplementary notes

Supplementary Note 1. Historical and contemporary studies assume that the effects on adult height sum up.

Since the dawn of biometry and genetics human height is portrayed as a trait that results from a sum of the individual contribution of different influences. This additivity assumption is explicitly made in early works and is justified by a "physiological" argument: "stature is not a simple element, but a sum of the accumulated lengths or thicknesses of more than a hundred bodily parts" (1). Additivity implies normality, and the adult height serves as an empirical example of a normally distributed biological trait in textbooks on statistics (2–4). Furthermore, an inheritance of human height is described by a model in which many genetic effects add up (additive polygenic model) (5,6). Indeed, up until now, no evidence for non-additive genetic interactions was found for height (7,8).

The "additive" view of adult height generates few expectations. First, according to the Pearson-Davin argument (9), unless the sizes of "bodily parts" of which the height is sum of are perfectly correlated, the coefficient of variation (CV, the ratio between the standard deviation, SD, and the mean) of height is expected to be low. This expectation is justified by the fact that the square of CV of a sum of random, strictly positive, variables is proportional to 1/**n**, where **n** is the number of variables; hence, as **n** grows, the CV decreases (10). Secondly, assumptions of the central limit theorem (11) are expected to be met for height (12), and its distribution in a population is expected to be normal. Indeed, both predictions hold rather well. The CV of within-sex adult height is in order of few percent. For more than a century, different studies assume adult height to be distributed normally (1,13); rather typically, investigators see that "any

transformation gives a slightly poorer fit in the case of height than none at all" (4). Thus, often the adult height serves as an empirical example of a normally distributed biological trait in textbooks on statistics (2–4). Thus, historically, adult height is described with an additive effects model.

A notable exception from this is the way some classical studies treat the effects of sex. Often, analyses are stratified by sex, such as in Pearson and Lee (14), while Galton (1) pre-adjusted the height for sex by multiplying female height by 1.08. Interestingly, Solomon et al. (15) formally demonstrate that for the effects of sex the "additive model is clearly rejected, but the multiplicative model provides an acceptable fit".

To characterise current practices in human height genetics, we performed a mini-review of a semi-random sample of literature. For that, we queried Google Scholar for "human height (genetic OR epidemiological) study" on Jan 28, 2020 using Internet Explorer web browser. We then analysed the top 50 articles that were retrieved. Results are presented in Supplementary Table 10.

Twenty-seven manuscripts published between 2003 and 2018 (median 2010) dealt with analysis of individual-level adult height data. The manuscripts were published in prestigious journals such as Nature, Nature Genetics, American Journal of Human Genetics and were jointly cited more than 5,000 times (minimum 7, maximum 1202, median – 54). Among these papers, only one work applied a non-linear transformation of height. Only 11 applied sex-stratified analysis or sex-specific standardization of height. A practice of sex-specific standardization and then joint analysis or meta-analysis is, in fact, similar to accounting for the effects of sex in a multiplicative (Galton's (1) and Thompson's (15)) manner.

Thus, the current dominating practice in the field of human height genetics is to treat all effects, including sex, as additive, and to assume normal distribution of the residuals. We should, however, note, that about 1/3 of studies agree with a classical tradition of special treatment of sex by performing sex-stratified Z-transformation.

Supplementary Note 2. Peculiarities in the current model of height.

From theory as well as from practice it is well-understood that normal distribution is only an approximation to the distribution of height in human populations. In fact, a scenario that would be the most favourable to a normal approximation would imply a compound-normal distribution of height, with total distribution being a mixture of normal distributions having different means in different sub-populations defined by sex, age, socio-economic status, and so forth. Thus, in fact, we do not assume normal distribution of height, but rather the normal distribution of height residuals.

Practically, we also notice several peculiarities in the “beautiful regularity in the statures of a population” (a quote from (1)), which may suggest that compound-normal may be not the best approximation either, — especially when we study large diverse populations.

The first peculiarity lies in an observation—coming from anthropometric and socioeconomic literature—that standard deviation of height tends to be greater in taller populations (16), while the coefficient of variation across populations is rather stable (see, e.g., Figure 2 from (17)). This low value and stability of the CV of adult height within human populations is rather noticeable when compared to the distribution of CV of weight (16) or to that of body length in other species (see Figure 2 of (18)). These observations led some social scientists and economists to postulate a log-normal distribution of height (19), implying a model under which effects multiply.

The second peculiarity relates to the way in which the field of quantitative genetics sometimes handles (a nuisance) effects of sex (covered briefly in Supplementary Note 1). In reasonably sized analysis groups one can observe that not only the height, but also

the standard deviation of height is larger in men than in women. Interestingly, the ratio between standard deviation of male and female height is close to the ratio between average male and female height. For future reference, we will call this observation "an approximate equality of SD and mean ratios between sexes". Several works, starting with Galton (1), dealt with sex differences in height by multiplying female height by ~1.08; and some works even formally demonstrated that the multiplicative adjustment for sex is statistically better than additive (15).

In some contemporary genetic studies the adjustment for the effects of sex is sometimes made by centering and scaling height in males and females separately, so that transformed height has the same mean and variance in both sexes (see Supplementary Note 1). The latter procedure is, in fact, very similar to the multiplicative adjustment, if we trust approximate equality of SD and mean ratios between sexes.

Several hypotheses may explain the observations summarised above, each of them having their own advantages and disadvantages. It may be that an additive model, that leads to normal approximation, is true. The greater variance in men's height may be explained in a number of ways. For example, the Geodakyan's theory (20) postulates a narrower norm of reaction in males, and hence, for a trait under stabilising selection in an outbred population, a larger total variance. Another broad explanation may be that the environmental effects on the stature are distributed differently for men and women, leading to the difference in the amount of environmentally determined variance. The apparent observation that the between-sex ratios in means and SDs are approximately equal—if indeed true—may be just a coincidence. Both these explanations, however, would also predict differences in heritability of height between males and females; however, this difference, if any, is very small (see, for example, (21,22)). We also feel that these explanations are unnecessary complex and a simpler explanation, when available, should be favoured.

Another explanation is that the multiplicative hypothesis, which would lead to a

log-normal approximation, may be true. However, in that case, why is the distribution of height so well described by the normal distribution (2–4), and why hasn't the log-normal distribution of height been detected before? If an additive (normal) model is used to study a trait that follows a multiplicative (log-normal) model, the groups defined by factors affecting the mean (e.g. a sex, socio-economic status, genotype) should exhibit different variance because of such property of a log-normal distribution as the scaling of standard deviation with the mean. Also, when a log-normally distributed trait is studied under a normal approximation, one would expect inclusion of multiplicative effects (i.e. epistatic and gene-environment interactions) to improve the fit of a model to the data (23), but this is not what we have seen up until now, at least for the genetic effects (see, e.g., (7)).

Finally, a "hybrid" hypothesis could be proposed, assuming that some effects multiply, while others add up. For example, the effects of sex and the factors that distinguish and generate height differences between different populations may multiply, consistent with the observations from anthropologic and socioeconomic studies and some practices of handling sex effects in human genetics. At the same time, the effects of other factors, such as genes, may add up.

Although the latter hypothesis can accommodate most observations, and, although implicitly, this hypothesis reflects the de-facto state of affairs, we believe that now it may be the time to explicitly formulate and revise our assumptions about the distribution of height. An explanation involving only additivity or only multiplicativity would be more internally consistent and parsimonious; and if the hybrid hypothesis is true, it would be interesting to see what distinguishes factors that act additively from these acting multiplicatively, and to try to understand the underlying biology.

The evidence supporting a multiplicative model and log-normal approximation of height mostly come from an anthropological comparison of variation of height between different populations, while the additive model is used in genetic studies of

between-individual variation within specific, homogeneous populations. Up until now, no data was available that would provide both the level of detail required by a genetic study and the variety and massive total amount of studied subjects as used by anthropological studies. This gap, however, was recently closed by the UK Biobank project (24).

Supplementary Note 3. Normal and log-normal approximations for height.

Under an additive (normal) model, height is expected to be a sum of many independent influences, i.e. $\mathbf{h = m + b_1 \cdot x_1 + b_2 \cdot x_2 + \ldots + b_n \cdot x_n} + \varepsilon$, where $\mathbf{m}$ is the offset, $\mathbf{b_i}$ is an effect of the ith factor, $\mathbf{x_i}$ is the value of the factor, and ε is an instance of the random error, that is assumed to be normally distributed. For example, if the factor in question is sex (coded with $\mathbf{x}$=1 for male and $\mathbf{x}$=0 for female), in Europeans, the effect $\mathbf{b}$ will be about 13 cm, because, on average, men are taller than women by 13 cm. Under a multiplicative (log-normal) model, $\mathbf{h = m \cdot b_1^{X1} \cdot b_2^{X2} \cdot \ldots \cdot b_n^{Xn} \cdot e^{\varepsilon}}$. In this model, the influence of sex may be expressed as $\mathbf{b}$ = 1.08, or that men are 8% taller than women. Thus, under an additive model, factors contribute constant absolute increments, while under a multiplicative model they contribute constant percentage points. Note that a multiplicative model becomes an additive model after taking a logarithm.

In absolute terms, a fixed percentage from a short base is smaller than the same percentage from a tall base. Hence, the multiplicativity (log-normality) hypothesis predicts that effect sizes and the standard deviation (SD) should increase proportionally to the group's mean. This scaling is expected to be highly systematic and predictable. If an effect size of some factor (or SD) is $\mathbf{b^0}$ in a group with the mean equal to $\mathbf{m^0}$, then in a group having mean of $\mathbf{m^*}$ the effect size (SD) will be $\mathbf{b^* = b^0 \cdot (m^* / m^0)}$. In other

words, the ratio between an effect and the group mean ($\mathbf{b}^* / \mathbf{m}^* = \mathbf{b}^0 / \mathbf{m}^0$), as well as the ratios of between-group effects (SDs) and between-group means ($\mathbf{b}^* / \mathbf{b}^0 = \mathbf{m}^* / \mathbf{m}^0$) is expected to be constant.

The interactions, epistasis, and variance heterogeneity we observed were highly regular, consistent with the multiplicativity hypothesis. For example, we see that the effect of genotype was 7.5% larger in men compared to women (in women, height changes by 2.13 cm as the polygenic height score changes by one SD; in men the change was 2.29 cm/SD, see Supplementary Table 6A). Similar scaling was observed for the effect of the residual predictor: 1.67 cm/SD in women and 1.81 cm/SD — that is 8.5% larger — in men. Not only the effects of the two predictors but also the SD of height in men was larger than the SD in women by a very similar fraction (8.7%, Supplementary Table 5A). Under the multiplicative model, the constant, 1.08 (8%), has a simple interpretation: an average man is taller than an average women on 8% ($\mathbf{(\ m^* / m^0\ )}$ = 176 cm / 163 cm = 1.08). Furthermore, consistent with a multiplicative model, we observed that the relative difference in SD and mean height values between two groups of people is similar, where the two groups are stratified by median polygenic height score (+2.2% for SD and +2.3% for the mean) or by median residual predictor (+1.4% for SD and +1.9% for the mean, Supplementary Table 5A).

As far as we can see, our discovery mainly has a conceptual value and may guide better interpretation of the results of analysis of big data. As we demonstrate, a study of big height data under an additive model demonstrated variance heterogeneity and interactions. Variance heterogeneity across genotypes is often interpreted as evidence of possible interactions (25–27) or environmental sensitivity (28); and each specific (e.g. gene by sex, gene-environment, gene-gene) interaction tend to be explained in terms of the underlying biology. In case of height, though, the variance heterogeneity and specific interactions we detected under additivity assumption are capturing the scale effects and are a consequence of the multiplicative nature of this trait. A study of

height on the multiplicative scale, using the log-normal distribution, is free from these artefacts. These conceptual results may have wider implications beyond the analysis of human height *per se*. We feel that a careful inspection of previous reports of (potential) interactions is warranted. When effect sizes and standard deviation correlate with the mean—as is the case for human height—a multiplicative model should be considered as a simple parsimonious explanation.

In biology, the log-normal distribution is ubiquitous (29,30). While examples of traits that are distributed log-normally are paramount, it is actually hard to name a trait that is believed to be distributed normally, with height being one of the very few but prominent examples. Some authors (31) argued that statistical analysis and reporting standards should change to account for the fact that most biological traits are multiplicative, and the most common distribution is log-normal. While we agree that, as a field, we may want to change our mind-set to consider log-normal as *a priori* most likely distribution, we appreciate the difficulty of reporting results on log-scale, or, more generally, the fact that a "scale transformation obscures rather than illuminates the description" (23). Presenting the results for multiplicative, log-normally distributed traits requires special methods (30). Although specifically for height, with its small CV, the difference between reporting results on arithmetic and geometric scale is often minimal, we explore different ways of presenting results obtained for (log)height in Supplementary Table 8.

To conclude, here we demonstrated that height is a trait, for which many effects appear to multiply. While in a homogenious population, after stratification by sex, the distribution of height is well approximated by a normal distribution, the log-normal approximation should be considered in analysis of big data, analysis of heterogeneous populations, and analysis of the extremes of height. Other variance-stabilising transformations may be considered as well (see Supplementary Note 2).

Still, the question of distribution of adult height is far from being solved. Although we

demonstrate that the effects sex, of genetic and sociodemographic factors multiply, and that overall, the log-normal approximation provides (slightly) better fit to the data, we see that both normal and log-normal approximations fail to model the distribution of height in its entirety (see Supplementary Note 5). The normal approximation fails to capture multiplicativity of effects and provides poor fit at both extremes of the distribution. The log-normal approximation provides a better fit at the right tail, but the deviation of the left tail from the expected still persists. Previously, it was hypothesised that the extreme short individuals may reflect the presence of rare alleles with large negative effects on height (32). We may also speculate that it could be that the adult height is indeed a mixture of multiplicative effects and some additive effects, e.g. technologically determined part of the measurement error.

Supplementary Note 4. Distribution and variance-stabilising transformation of big height data.

While in this work we used simple logarithmic transformation, a range of other variance-stabilising transformation should work equally well when analysing adult height. For example, we tested how linear and log-transformation compare using the Box-Cox approach and found that parameter lambda=0 (log-transformation) results in log-likelihood (LL) of -3.015.614, while lambda=1 (equivalent to no transformation) gives a log-likelihood of -3.016.275. Interestingly, the maximum appeared at lambda=0.2 with LL=-3.015.571. In fact, one can obtain a distribution that would maximise lambda at ~0.2 by adding a small additive noise (SD ~ 7mm) on the top of a log-normal distribution mimicking the distribution of adult height.

In genome-wide association studies (GWAS) of "omics" data, traits are numerous

(hundreds and even tens of thousands), and often distributed neither normally nor log-normally. This led to a practice of applying quantile transformation to normality (often also called inverse-normal transformation) that, in absence of ties, leads to perfect normal distribution. One usual option is to first obtain residuals from linear regression of the trait onto a set of fixed covariates (usually sex, age; often principal components of genomic kinship matrix), perform quantile transformation of the residuals to normality and then running GWAS that estimates additive effect a SNP onto transformed residuals. Alternative protocol assumes quantile transformation to normality first, and running GWAS model that jointly estimates the additive SNP effect and the effects of covariates. When the effects of covariates and genetic polymorphisms are small, the above procedures work satisfactory in practical terms. However, neither procedure is theoretically satisfactory, and provides unsatisfactory results when effects become bigger.

This can be understood from the following example: consider a distribution a log-normally distributed trait y, so that

log(y) = mu + B1*g + B2*sex + error

Where “error” is a normally distributed error and B1 is an effect of a genotype and B2 is an effect of sex. It is quite obvious that if one takes log(y) and then runs a linear model adjusting for g and sex, the residuals from this model will be distributed normally. When one, however, would first perform quantile normalisation (inverse-normalisation) of y, and then adjust for g and sex; or would pre-adjust for sex, then perform quantile normalisation, and then account for the effect of g, the residuals from either of the procedures will not be distributed normally.

As an example, a reader can run the following code in R:

```
# model log-normally distributed trait
set.seed(2)
N=1000; q=0.4
g=rbinom(N,2,q); sex=rbinom(N,1,0.5);x=exp(rnorm(N,m=g+2*sex,sd=0.5))
# proper transformation
x1=log(x);
# inverse-normal variant 1
x2=qnorm((rank(resid(lm(x~sex)) , na.last="keep")-0.5)/sum(!is.na(x)))
# inverse-normal variant 2
x3=qnorm((rank(x,na.last="keep")-0.5)/sum(!is.na(x)))
r1=resid(lm(x1~g+sex));r2=resid(lm(x2~g));r3=resid(lm(x3~g+sex))
shapiro.test(r1); shapiro.test(r2); shapiro.test(r3);
```

That results in p=0.8, 4e-5 and 3e-5 for the logarithmic transformation, and inverse-normal transformation of type 1 and 2, respectively.

Supplementary figures

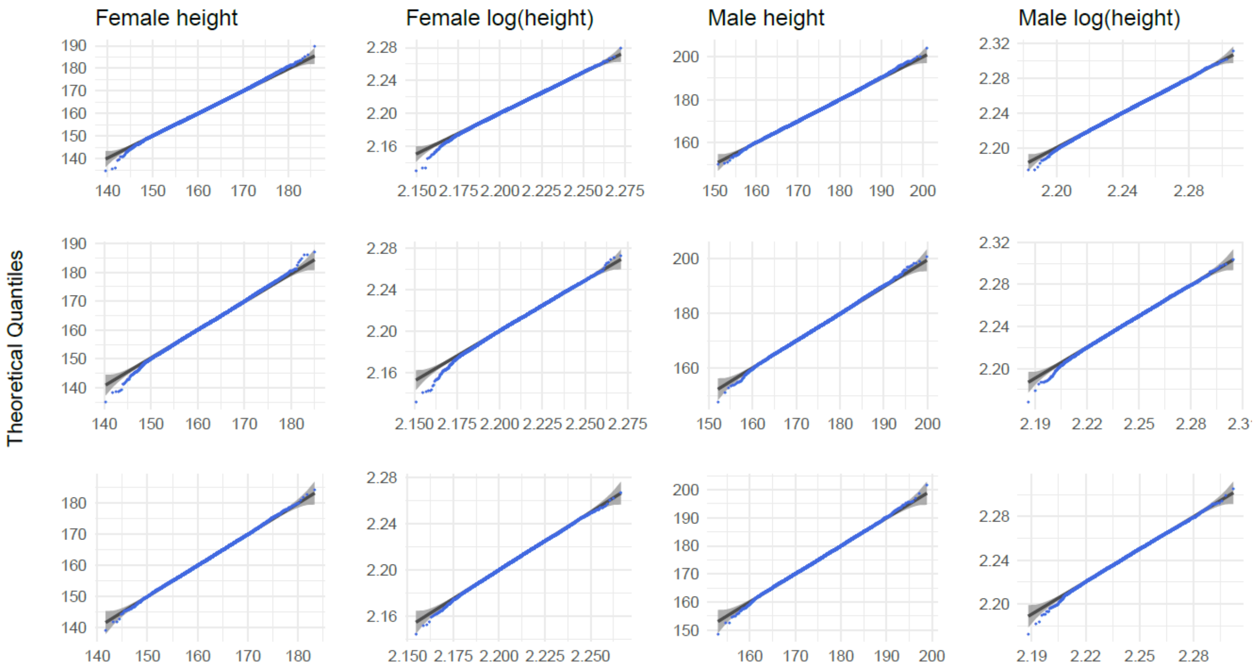

Supplementary Figure 1.

Q-Q plots of distributions of sex-stratified residuals adjusted for RP and PGHS for European ancestry British born in different regions. Data shown only for the three major populations. Grey areas correspond to the 95% CI.

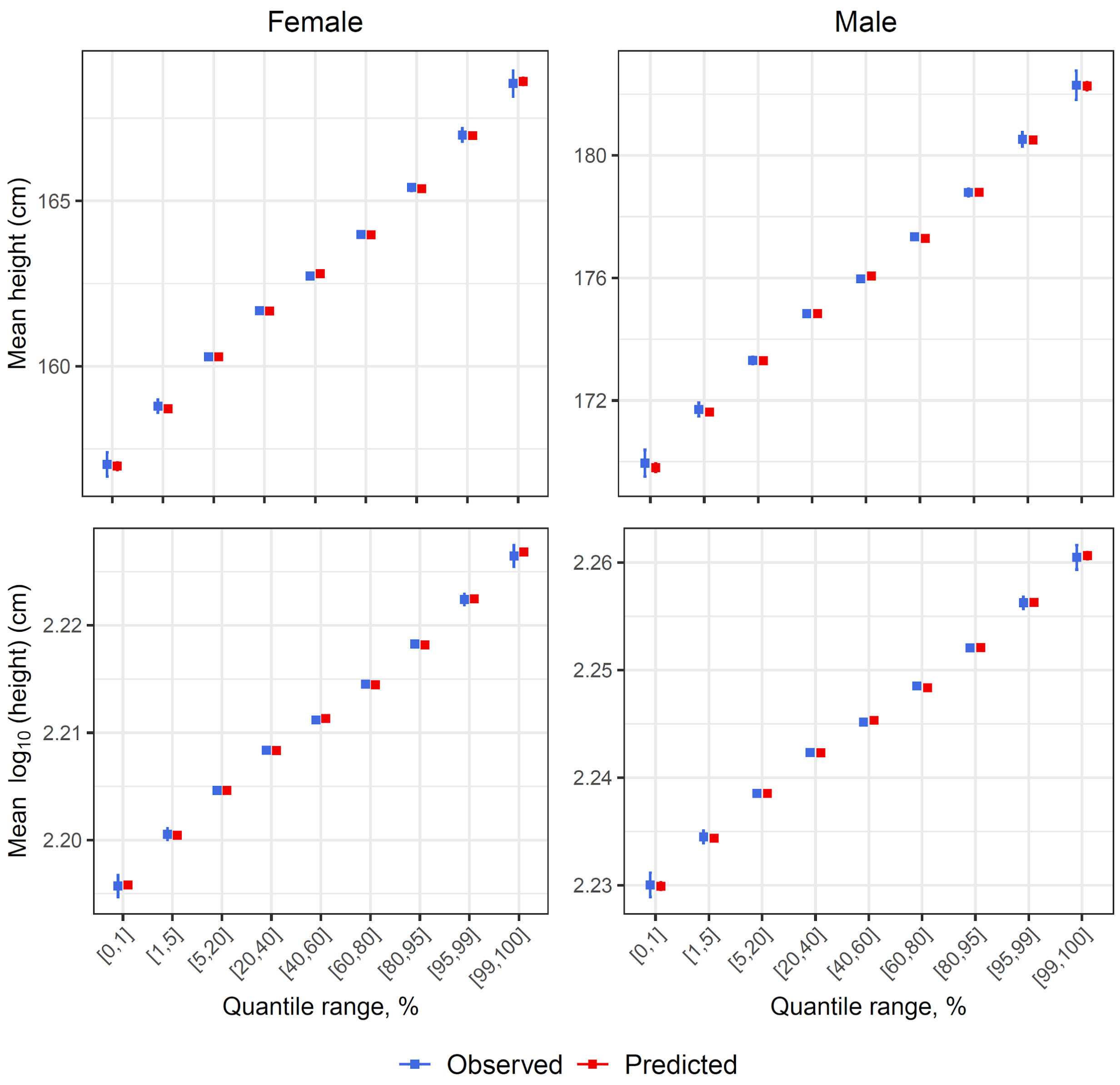

Supplementary Figure 2.

Observed (green) and predicted (red) mean male and female height and log-height with PGHS and RP as predictors in English. Each point represents the mean value of observed or predicted height values for samples from several distribution ranges. Error Bars show 95% CI for the estimate of the mean.

References

1. Galton F. Regression Towards Mediocrity in Hereditary Stature. The Journal of the Anthropological Institute of Great Britain and Ireland. 1886;15:246–63.

2. Snedecor GW. Statistical Methods: By George W. Snedecor and William G. Cochran. Iwoa State University Press; 1989. 503 p.

3. Devore JL, Berk KN. Modern Mathematical Statistics with Applications. Springer, New York, NY; 2012.

4. Wright S. Evolution and the genetics of populations. Vol. 1. Genetic and biométrie foundations. London and Chicago: University of Chicago Press.; 1968.

5. Fisher RA. XV.—The Correlation between Relatives on the Supposition of Mendelian Inheritance. Earth Environ Sci Trans R Soc Edinb. 1918;52(2):399–433.

6. Visscher PM. Sizing up human height variation. Nat Genet. 2008 May;40(5):489–90.

7. Aulchenko YS, Struchalin MV, Belonogova NM, Axenovich TI, Weedon MN, Hofman A, et al. Predicting human height by Victorian and genomic methods. Eur J Hum Genet. 2009 Aug;17(8):1070–5.

8. Visscher PM, McEvoy B, Yang J. From Galton to GWAS: quantitative genetics of human height. Genet Res . 2010 Dec;92(5-6):371–9.

9. Pearson K, Davin AG. On the Biometric Constants of the Human Skull. Biometrika. 1924;16(3/4):328–63.

10. Landau LD, Livshits EM. Statistical physics (in Russian). Gosudarstv. Izdat. Tehn.-Teor. Lit., Moscow; 1938.

11. Ljapunov AM. Nouvelle forme du théoreme sur la limite de probabilité. 1901.

12. Bland JM, Altman DG. Transforming data. BMJ. 1996 Mar 23;312(7033):770.

13. Galton Francis. XII. The geometric mean, in vital and social statistics. Proc R Soc Lond. 1879 Jan 1;29(196-199):365–7.

14. Pearson K, Lee A. On the Laws of Inheritance in Man: I. Inheritance of Physical Characters. Biometrika. 1903;2(4):357–462.

15. Solomon PJ, Thompson EA, Rissanen A. The inheritance of height in a Finnish population. Ann Hum Biol. 1983 May;10(3):247–56.

16. Schmitt LH, Harrison GA. Patterns in the within-population variability of stature and weight. Ann Hum Biol. 1988 Sep;15(5):353–64.

17. Blum M. Estimating male and female height inequality. Econ Hum Biol. 2014 Jul;14:103–8.

18. McKellar AE, Hendry AP. How Humans Differ from Other Animals in Their Levels of Morphological Variation [Internet]. Vol. 4, PLoS ONE. 2009. p. e6876. Available from: http://dx.doi.org/10.1371/journal.pone.0006876

19. Soltow L. Inequalities in the Standard of Living in the United States,1798-1875. In: American Economic Growth and Standards of Living before the Civil War. University of Chicago Press; 1992. p. 121–72.

20. Geodakyan VA. Differential mortality and the norm of reaction of males and females (in Russian). Zhurnal Obshey Biologii. 1974;35(3):376–85.

21. Rawlik K, Canela-Xandri O, Tenesa A. Evidence for sex-specific genetic architectures across a spectrum of human complex traits. Genome Biol. 2016 Jul 29;17(1):166.

22. Zillikens MC, Yazdanpanah M, Pardo LM, Rivadeneira F, Aulchenko YS, Oostra BA, et al. Sex-specific genetic effects influence variation in body composition. Diabetologia. 2008 Dec;51(12):2233–41.

23. Falconer DS, Mackay TFC. Introduction to quantitative genetics. 1996.

24. Bycroft C, Freeman C, Petkova D, Band G, Elliott LT, Sharp K, et al. The UK Biobank resource with deep phenotyping and genomic data. Nature. 2018 Oct;562(7726):203–9.

25. Paré G, Cook NR, Ridker PM, Chasman DI. On the use of variance per genotype as a tool to identify quantitative trait interaction effects: a report from the Women's Genome Health Study. PLoS Genet. 2010 Jun 17;6(6):e1000981.

26. Struchalin MV, Dehghan A, Witteman JC, van Duijn C, Aulchenko YS. Variance heterogeneity analysis for detection of potentially interacting genetic loci: method and its limitations. BMC Genet. 2010 Oct 13;11:92.

27. Wang H, Zhang F, Zeng J, Wu Y, Kemper KE, Xue A, et al. Genotype-by-environment interactions inferred from genetic effects on phenotypic variability in the UK Biobank. Science Advances. 2019 Aug 1;5(8):eaaw3538.

28. Yang J, Loos RJF, Powell JE, Medland SE, Speliotes EK, Chasman DI, et al. FTO genotype is associated with phenotypic variability of body mass index. Nature. 2012 Oct 11;490(7419):267–72.

29. Koch AL. The logarithm in biology. J Theor Biol. 1969;23(2):251–68.

30. Limpert E, Stahel WA, Abbt M. Log-normal distributions across the sciences: keys and clues. Bioscience. 2001;51(5):341–52.

31. Limpert E, Stahel WA. Problems with using the normal distribution--and ways to improve quality and efficiency of data analysis. PLoS One [Internet]. 2011;6(7). Available from: https://www.ncbi.nlm.nih.gov/pmc/articles/pmc3136454/

32. Chan Y, Holmen OL, Dauber A, Vatten L, Havulinna AS, Skorpen F, et al. Common variants show predicted polygenic effects on height in the tails of the

distribution, except in extremely short individuals. PLoS Genet. 2011 Dec;7(12):e1002439.
